# Supplementary material for: Analysis of 3D genomic interactions identifies candidate host genes that transposable elements potentially regulate
Source: Genome Biol. 2018 Dec 13;19:216. doi: 10.1186/s13059-018-1598-7 (PMC6292174; doi:10.1186/s13059-018-1598-7)
Supplement: Supplementary file 1 — Supplementary Figures. This file contains all the supplementary figures for this paper. (PDF 4494 kb) [file 13059_2018_1598_MOESM1_ESM.pdf]

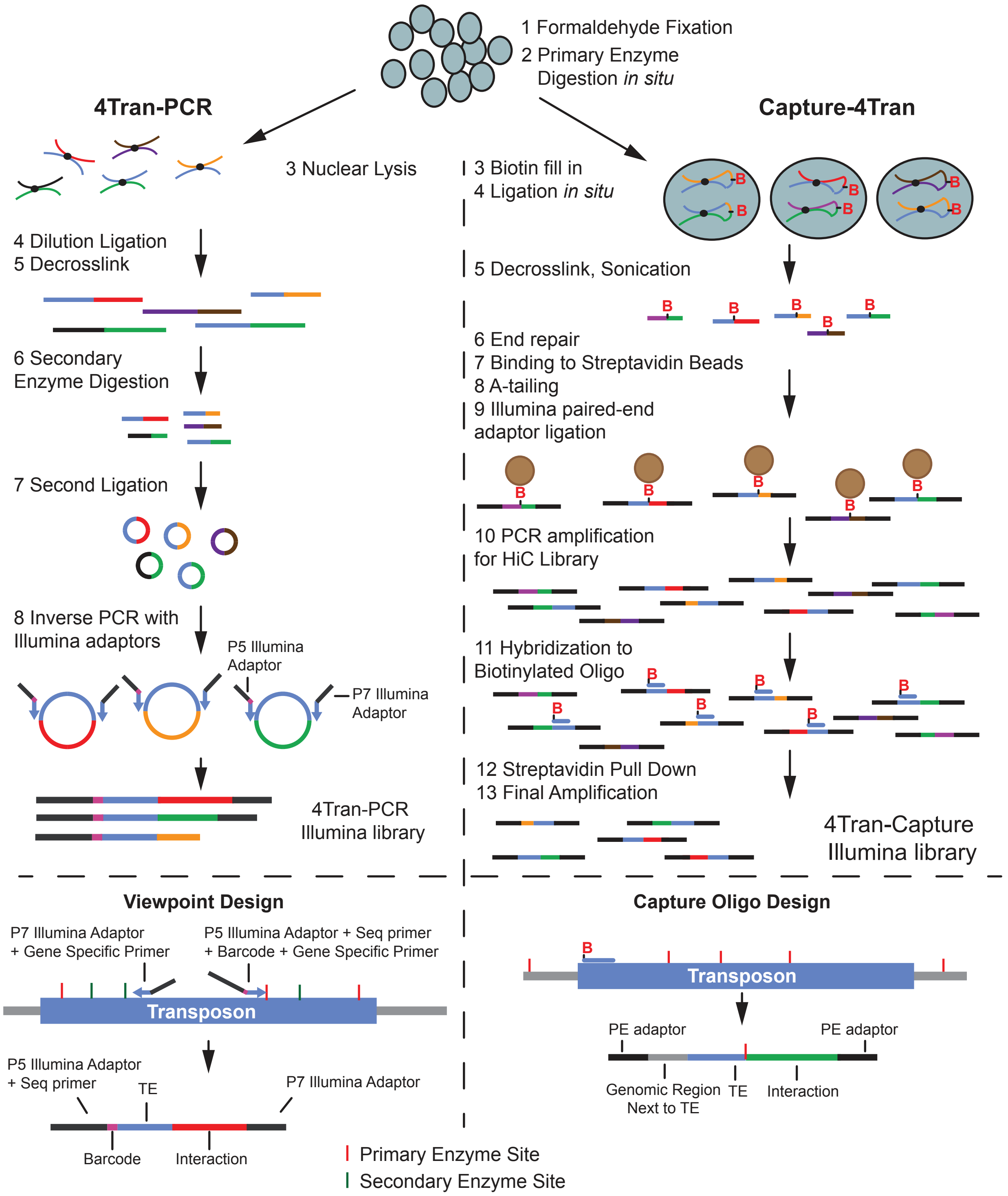
1 Formaldehyde Fixation
2 Primary Enzyme Digestion *in situ*
4Tran-PCR
Capture-4Tran
3 Nuclear Lysis
3 Biotin fill in
4 Ligation *in situ*
B
4 Dilution Ligation
5 Decrosslink
5 Decrosslink, Sonication
6 Secondary Enzyme Digestion
6 End repair
7 Binding to Streptavidin Beads
8 A-tailing
9 Illumina paired-end adaptor ligation
7 Second Ligation
10 PCR amplification for HiC Library
8 Inverse PCR with Illumina adaptors
P5 Illumina Adaptor
P7 Illumina Adaptor
11 Hybridization to Biotinylated Oligo
12 Streptavidin Pull Down
13 Final Amplification
4Tran-PCR Illumina library
4Tran-Capture Illumina library
Viewpoint Design
Capture Oligo Design
P7 Illumina Adaptor + Gene Specific Primer
P5 Illumina Adaptor + Seq primer + Barcode + Gene Specific Primer
Transposon
Transposon
PE adaptor
PE adaptor
Genomic Region Next to TE
TE
Interaction
P5 Illumina Adaptor + Seq primer
TE
P7 Illumina Adaptor
Barcode
Interaction
Primary Enzyme Site
Secondary Enzyme Site

Figure S1

Fig. S1- Schematic representation of 4Tran-PCR and 4Tran-Capture approaches. The protocol for 4Tran-PCR is the same as described in [90] for both template preparation and library amplification. Although not shown here, we also successfully tested in situ ligation by simply omitting the SDS treatment step following digestion with the primary restriction enzyme. Scheme shows amplification using Illumina single end reads. The protocol also works with paired-end adaptors and for newer Illumina machines these paired-end adaptors are necessary. Capture-4Tran is similar to Hi-C and Capture-C protocols. Our strategy to identify both an interacting fragment and a specific TE integration consists of designing a probe close to the 5’ or 3’ end of the transposon. With this strategy most reads will contain an interaction fragment, part of the transposon to which the oligonucleotide probe hybridizes and the genomic region immediately adjacent to the integration event that can identify one side of an interaction containing the TE.

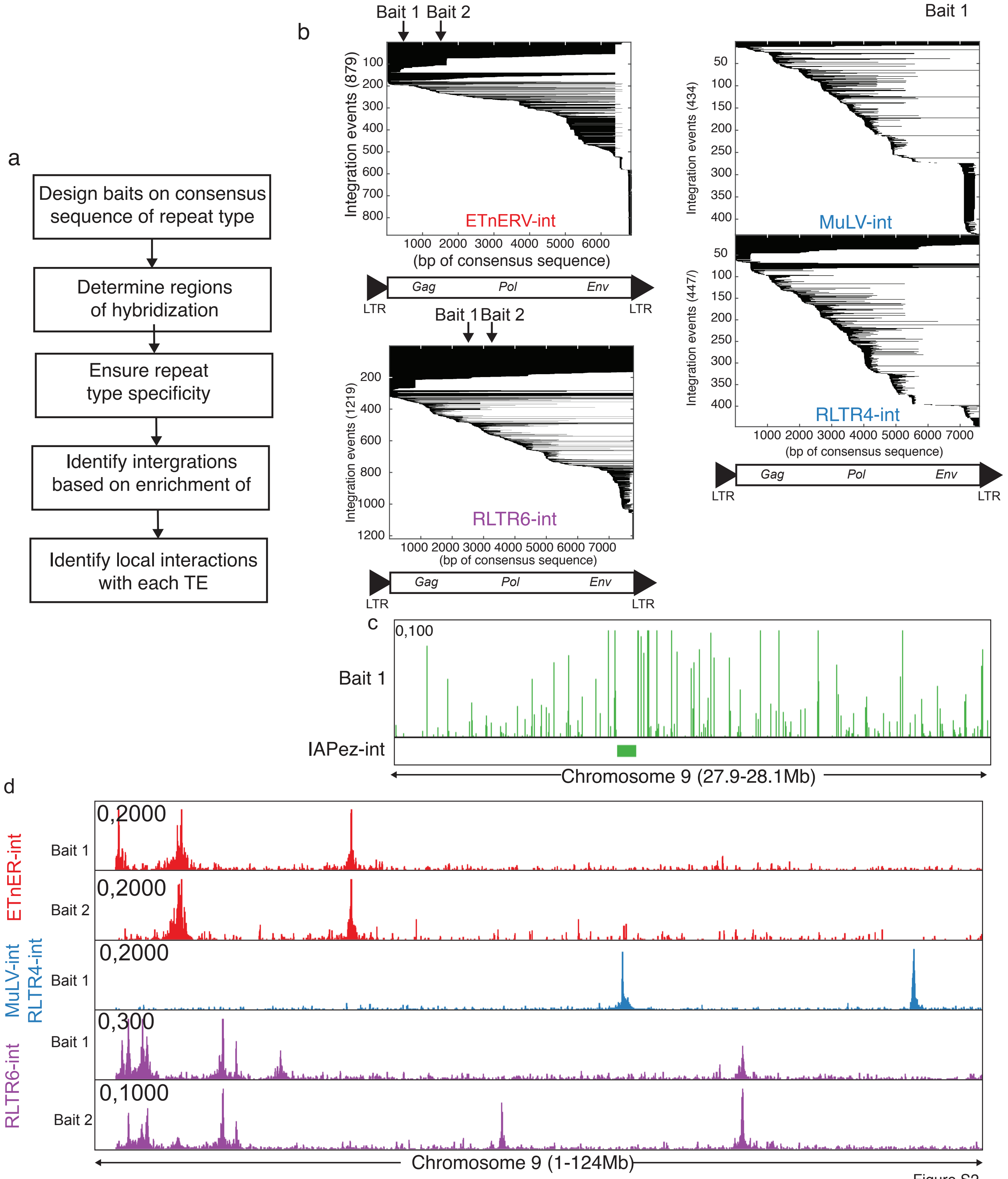

a
Design baits on consensus sequence of repeat type
Determine regions of hybridization
Ensure repeat type specificity
Identify intergrations based on enrichment of
Identify local interactions with each TE
b
Bait 1 Bait 2
Integration events (879)
ETnERV-int
(bp of consensus sequence)
Gag Pol Env
LTR LTR
Bait 1
Integration events (434)
MuLV-int
Integration events (447/)
RLTR4-int
Integration events (1219)
RLTR6-int
c
0,100
Bait 1
IAPez-int
Chromosome 9 (27.9-28.1Mb)
d
ETnER-int
MuLV-int RLTR4-int
RLTR6-int
Bait 1
Bait 2
0,2000
0,300
0,1000
Chromosome 9 (1-124Mb)

Figure S2

Fig.S2- a Workflow for 4Tran-PCR design and analysis. b Schematic representation of all integration events ERVs shown. Each line represents a different integration and the black shows which part of the consensus sequence (shown under the plot) is retained. Integration events are sorted by 5' position on the consensus sequence and then by size of integration. Arrows represent the location tested for 4Tran-PCR baits. c Zoom in of a region on chromosome 9 showing raw 4Tran-PCR signal around an IAPEz integration (shown as a bar under the plot). d Raw 4Tran-PCR data for the baits tested in mES cells.

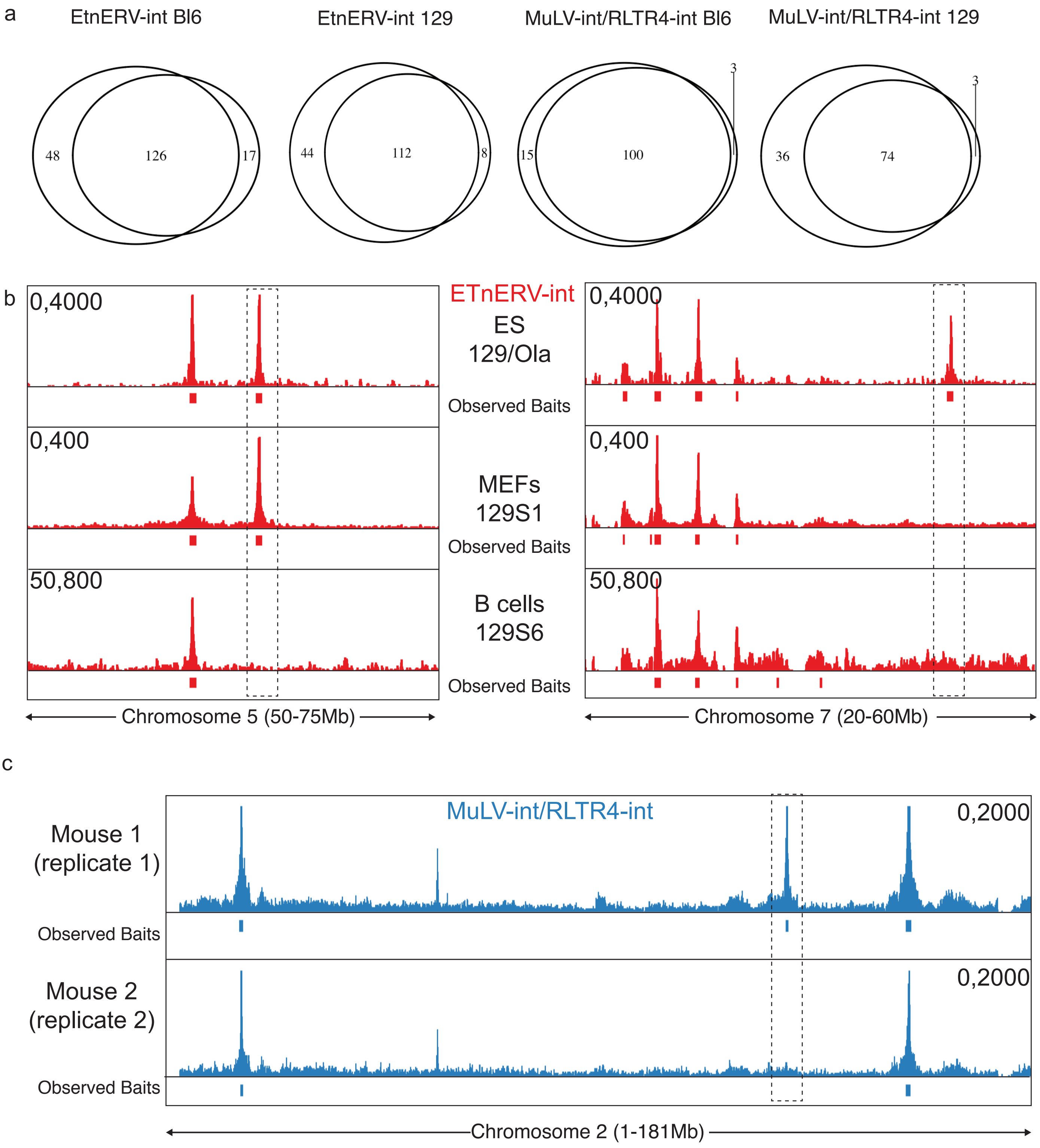
a
EtnERV-int Bl6
48
126
17
EtnERV-int 129
44
112
8
MuLV-int/RLTR4-int Bl6
3
15
100
MuLV-int/RLTR4-int 129
3
36
74
b
ETnERV-int
0,4000
ES
129/Ola
Observed Baits
0,400
MEFs
129S1
Observed Baits
50,800
B cells
129S6
Observed Baits
Chromosome 5 (50-75Mb)
Chromosome 7 (20-60Mb)
c
MuLV-int/RLTR4-int
Mouse 1
(replicate 1)
0,2000
Observed Baits
Mouse 2
(replicate 2)
0,2000
Observed Baits
Chromosome 2 (1-181Mb)

Figure S3

Fig.S3 – a Overlap of observed bait calls between replicates. b Examples of shared and different integrations of ETnERV elements. The same primer pair was used to amplify signal from either mouse embryonic stem cells of the 129/Ola substrain, mouse embryonic fibroblast from the 129S1 substrain and splenic B cells of the 129S6 substrain. The locations of observed bait-like profiles is shown under the plots. c Example of a different integrations site detected in littermates.

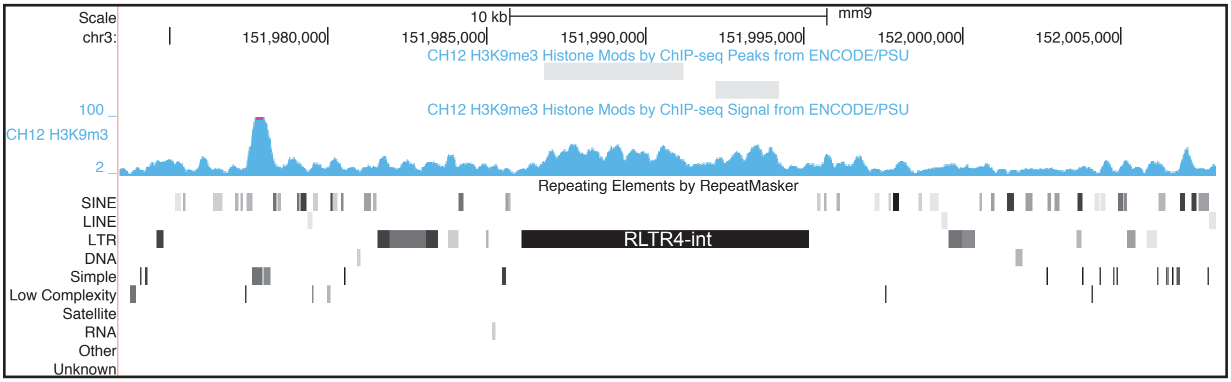

Fig.S4 - UCSC browser view of mouse chromosome 3 and of the ENCODE data for H3K9me Chip-Seq in the region surrounding the RLTR4 integration shown in Figure 3

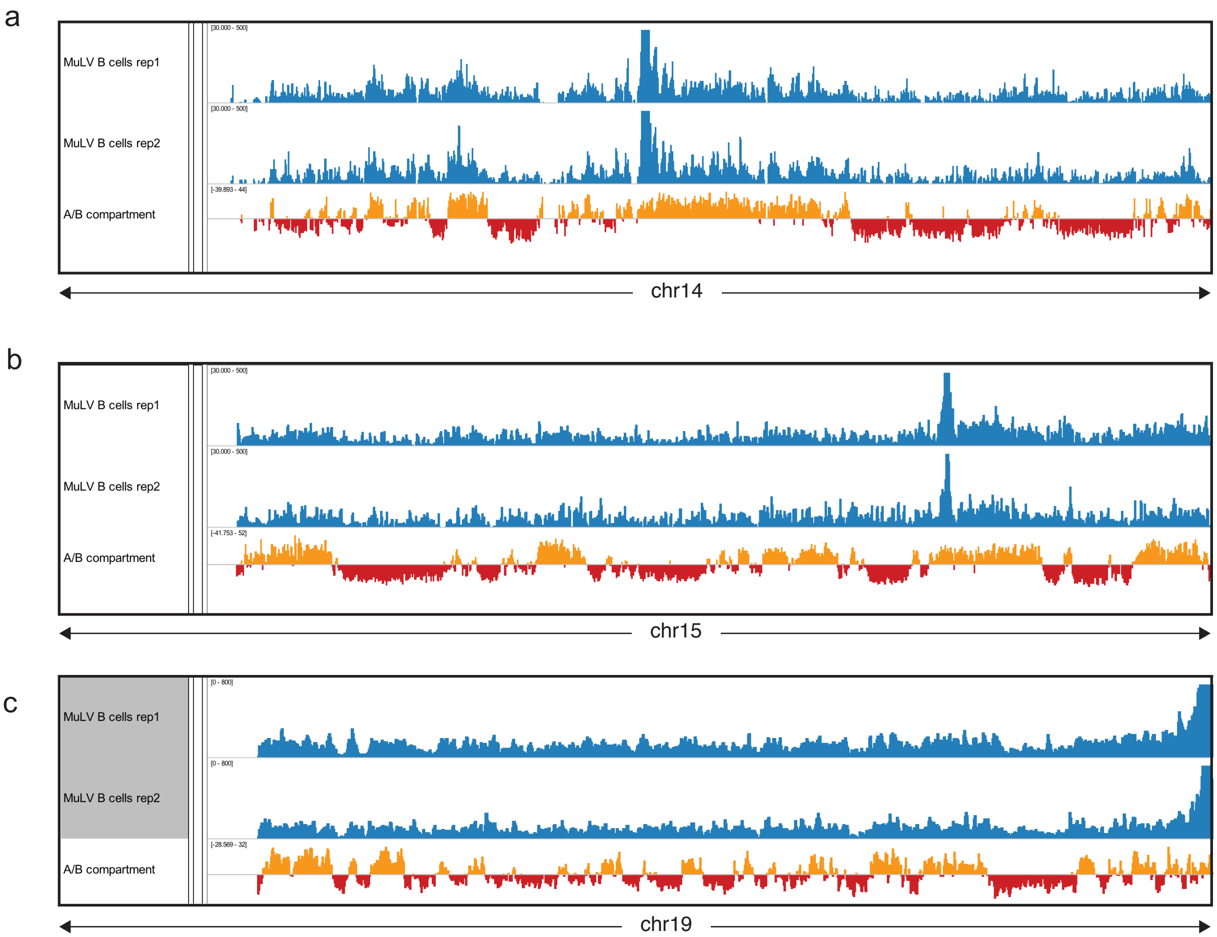

Fig.S5 – a,b,c Whole chromosome browser view of 4C-Seq signal and PC scores from Hi-C.

a
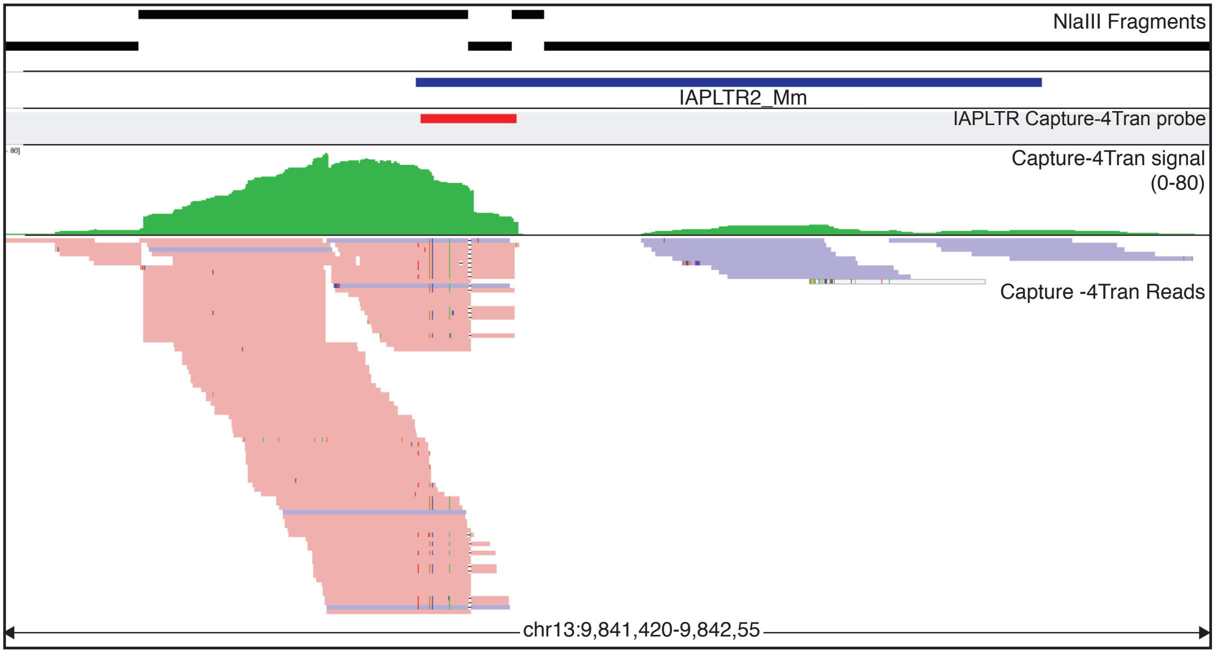

b
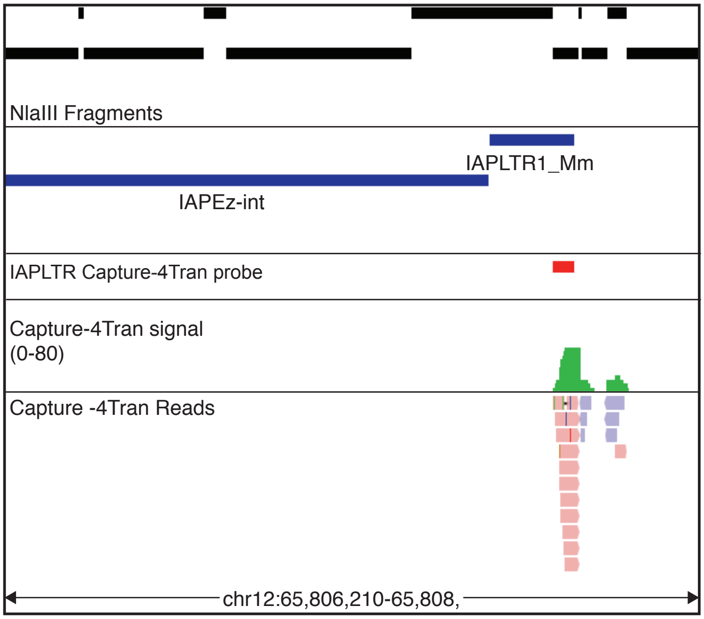

Fig.S6 – Browser view of Capture-4Tran using an IAPLTR probe on a solo LTR (a) and surrounding a full length IAPEz (b). Top track represents the DNA fragments generated by NlaIII digestion. The location of ERV elements followed by predicted and detected integration sites shown respectively as a peak profile or with actual location of reads is shown below.

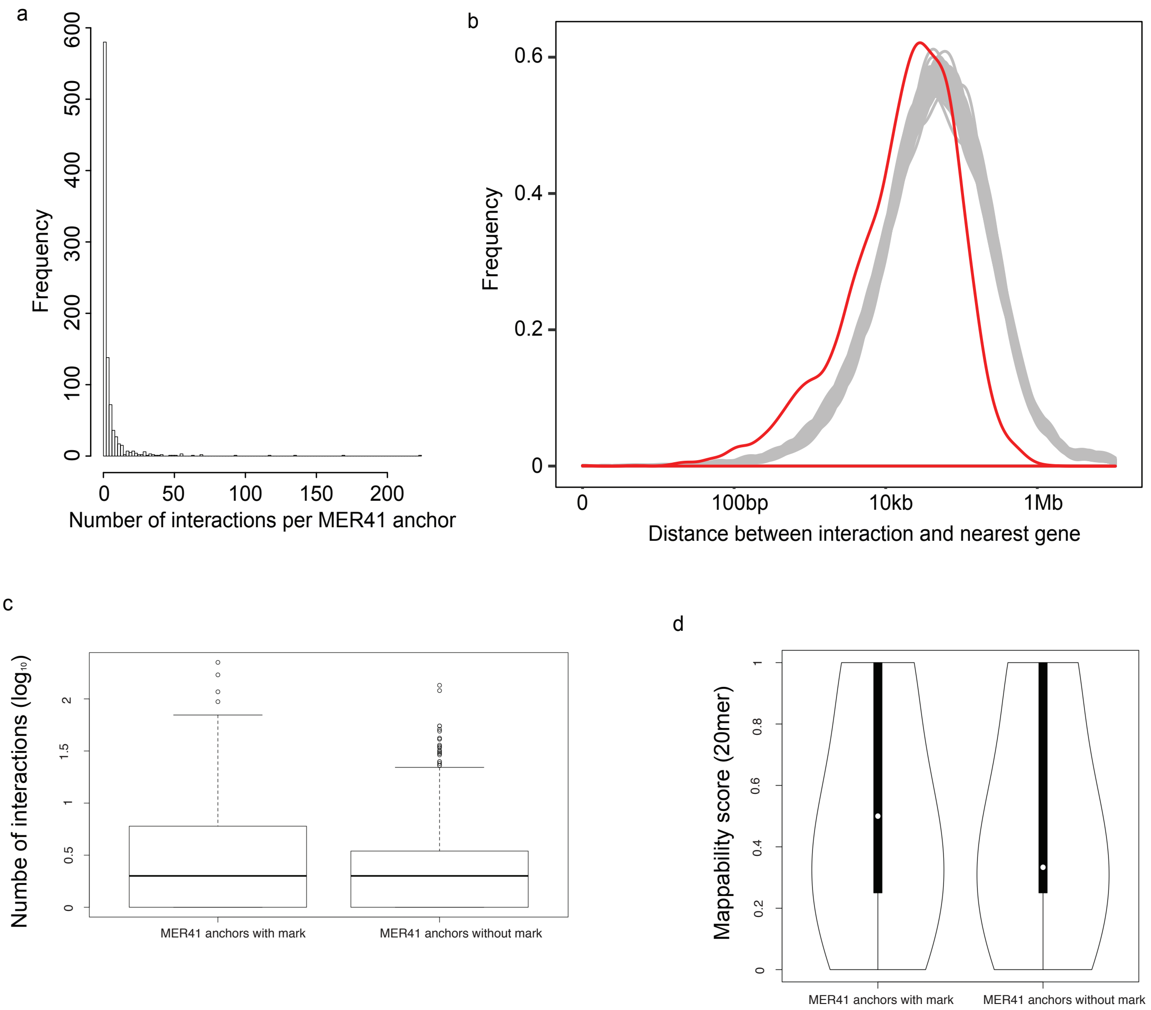

e

Interactions between MER41 elements

	Interaction		
Capture-bait	MER41A	MER41B	MER41C
MER41A	8	4	1
MER41B	3	15	0

Fig.S7 – a Histogram of the number of interactions from each MER41 anchor. b Distance between MER41 element to nearest gene compared to a random set of regions. c Boxplot of the interactions from MER41 baits that overlap with a ChIP-Seq peaks and MER41 baits that do not overlap with a ChIP-Seq. d Mappability score of 20mer sequences that fall within MER41 baits that overlap with a ChIP-Seq peaks and MER41 baits that do not overlap with a ChIP-Seq. e Number of interactions from MER41A and MER41B anchors that overlap with any MER41 element.

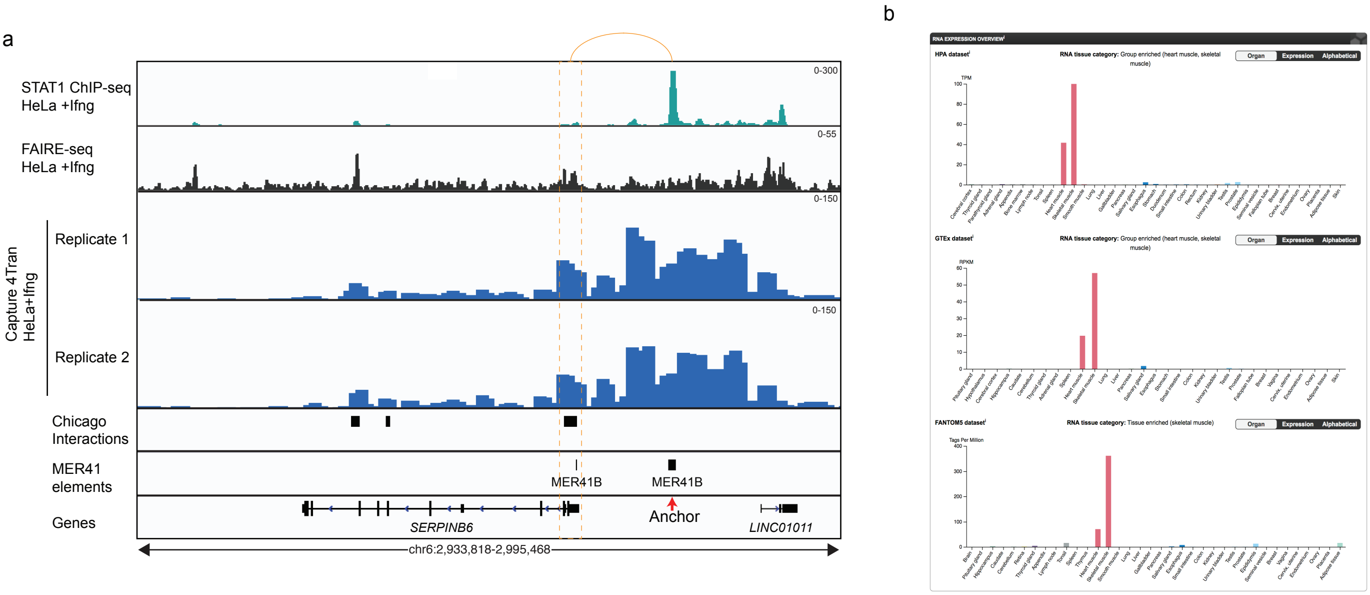

Fig. S8 – a Genomic tracks of ChIP-Seq signal and 4Tran data from control and IFNγ treated HeLa cells. Red arrow represents the MER41B anchor and the orange dotted rectangle represents the interactions. b Screenshot of tissue specific expression of MYPN gene from Human Protein Atlas.
